# Influence of resin-modified glass ionomer and topical fluoride on levels of *Streptococcus mutans* in saliva and biofilm adjacent to metallic brackets

**DOI:** 10.1590/1678-77572016-0231

**Published:** 2017

**Authors:** Marcela Cristina Damião ANDRUCIOLI, Gisele FARIA, Paulo NELSON-FILHO, Fábio Lourenço ROMANO, Mírian Aiko Nakane MATSUMOTO

**Affiliations:** 1 Universidade de São Paulo, Faculdade de Odontologia de Ribeirão Preto, Departamento de Clínica Infantil, Ribeirão Preto, SP, Brasil.; 2 Universidade Estadual Paulista, Faculdade de Odontologia de Araraquara, Departamento de Odontologia Restauradora, Araraquara, SP, Brasil.

**Keywords:** Orthodontics, Glass ionomer cements, Streptococcus mutans, Acidulated phosphate fluoride, Dental materials

## Abstract

Decalcification of enamel during fixed orthodontic appliance treatment remains a problem. White spot lesions are observed in nearly 50% of patients undergoing orthodontic treatment. The use of fluoride-containing orthodontic materials has shown inconclusive results on their ability to reduce decalcification. The aims of this investigation were to compare the levels of *Streptococcus mutans* (SM) in saliva and biofilm adjacent to orthodontic brackets retained with a resin-modified glass ionomer cement (RMGIC) (Fuji ORTHO LC) and a light cured composite resin (Transbond XT), and to analyze the influence of topical application of the 1.23% acidulated phosphate fluoride (APF) on SM counts. In a parallel study design, two groups (n=14/15) were used with random allocation and high salivary SM counts before treatment. Biofilm was collected from areas adjacent to the brackets on teeth 13, 22, 33, and 41. Both saliva and biofilm were collected on the 7^th^, 21^st^, 35^th^, and 49^th^ days after appliance placement. Topical fluoride application was carried out on the 35^th^ day. Bonding with RMGIC did not alter SM counts in saliva or biofilm adjacent to the brackets. On the other hand, the biofilm adjacent to brackets retained with composite resin showed a significant increase in SM counts along the trial period. Topical application of 1.23% APF did not reduce salivary or biofilm SM counts regardless of the bonding material. In conclusion, fluoride topical application did not show efficacy in reducing SM. The use of RMGIC as bonding materials allowed a better control of SM cfu counts in dental biofilm hindering the significant increase of these microorganisms along the trial period, which was observed in the biofilm adjacent to the composite material.

## Introduction

Decalcification around orthodontic brackets is a common problem and a potential risk of orthodontic treatment, especially in patients with poor oral hygiene^13,23^. There is a significant increase in salivary and biofilm levels of *Streptococcus mutans* (SM) in nearly 50% of patients undergoing fixed orthodontic treatment, concomitant with an increased risk to dental caries^2,14,17^. Fixed orthodontic appliances induce intraoral changes, such as a low-pH environment, which increase enamel susceptibility to the formation of white spot lesions (WSL) caused by organic acids produced by dental biofilm bacteria. WSL are potentially irreversible and can be observed as early as 1 month after the beginning of orthodontic treatment, causing aesthetic problems^1,3,11,12^.

An effective biofilm control is the main measure to prevent enamel decalcification in orthodontic patients, but it depends on patient compliance^4^ and thus, other preventive measures such as topical fluoride should be associated^2,8,17^. There are several forms of delivering topical fluoride during orthodontic treatment, which include toothpastes, mouthrinses, gel, varnishes, fluoride-releasing materials, such as bonding materials and elastics^1,3,4,24^.

The use of glass ionomer cement (GIC) for bracket bonding is a mean to release fluoride from a rechargeable source to sites at higher risk for developing dental caries^1,3,4,9,11,12,19,24,25^. Resin modified GIC (RMGIC) is a good alternative for orthodontic bonding as they offer protection against demineralization by fluoride release and present higher resistance to bracket debonding than conventional GIC^1,5,6,14,15,21,22,24,30^.

The antimicrobial activity of GIC is due to a combination of fluoride release from the cements and decrease of pH during the setting reaction, which increases the sensitivity of microorganisms to fluoride^7,14,28^. Furthermore, the literature shows that fluoride recharging restore the antibacterial properties of the resin-modified glass ionomer cements^27^.

The influence of GIC on the control of SM counts in saliva and biofilm has been extensively evaluated in clinical trials. However, these studies have employed the split-mouth design whereby the test material is allocated to two quadrants of the mouth and the control material is allocated to the other two quadrants at the same time^11,16,18,29^. This trial design can lead to same cross-over effect onto the control side.

The aims of this parallel designed clinical trial was to compare the effect of two orthodontic bonding materials (RMGIC and composite resin) on the levels of SM in saliva and biofilm adjacent to orthodontic brackets and to evaluate if a 1.23% acidulated phosphate fluoride gel application can influence the reduction of SM counts.

## Material and methods

Eligible participants were screened among patients with indication for orthodontic treatment with fixed appliances, who had complete permanent dentition, were free of caries, had good general health, and had not used antibiotics and/or antimicrobial mouthwashes in the previous 3 months. The minimum sample size for each group (with 80% power and a significance level of 5%) was identified as 14 for an expected difference of 30% between the groups. Twenty-nine 12-20-year-old patients who met the inclusion criteria and presented high salivary SM counts were enrolled in the study. Following the approval of the research project by the institutional Ethics Committee (process #2005.1.1013.58.7), all subjects or their parents/caregivers received verbal and written information about the study purposes and procedures and signed an informed consent form for participation. All patients were resident in an area with fluoridation of the public water supply. Biofilm deposits were eliminated with meticulous rubber cup/pumice prophylaxis, and patients received general oral hygiene instructions and were oriented to brush their teeth 3 times a day after meals using a toothbrush (Professional^®^, Colgate-Palmolive Indústria and Comércio Ltda., São Paulo, SP, Brazil) and a fluoride-containing dentifrice (Colgate Máxima Proteção Anticaries^®^, Colgate-Palmolive Indústria and Comércio Ltda., São Paulo, SP, Brazil) supplied by the researchers throughout the experimental period. No other fluoride sources were used.

Before the beginning of the study, the number of SM colony-forming units (cfu) in the saliva of all eligible individuals was determined. For this purpose, 2.0 mL of non-stimulated saliva were collected from each patient before placement of the appliance (T_0_). The material collected was placed in properly labeled 15x100 mm sterile tubes containing 4 to 5 glass beads. Aliquots of 0.05 mL of diluted pure non-stimulated saliva samples were seeded on SB-20M solid culture medium, prepared according to Saravia, et al.^26^(2013). The number of colony forming units (cfu) *per* milliliter of saliva was counted under aseptic conditions under a stereomicroscope (Nikon, Tokyo, Japan) with reflected light. Colonies with MS characteristics were transferred to tubes containing thioglycollate (Difco Laboratories Inc., Detroit, MI, USA) and incubated at 37^o^C during 24 h for biotyping. The growth of MS cfu was verified after the incubation period, and the following tests were performed for biochemical identification: fermentation of mannitol, sorbitol, raffinose and melibiose, resistance to bacitracin, hydrolysis of arginine and sculin, production of H_2_O_2_, and sensitivity to 2.0 IU bacitracin.

In all patients, sterile new Edgewise metallic orthodontic brackets (0.022x0.028-inch slot) (Generus, GAC International Inc., Bohemia, NY, USA) were bonded in all teeth with a RMGIC (Fuji Ortho LC; CG Corporation, Tokyo, Japan - experimental group, n=14) or an orthodontic light-cured composite resin (Transbond XT, 3M Unitek, Monrovia, CA, USA - control group, n=15). The 29 patients were randomized to the two groups using the SAS (Statistical Analysis Systems) version 9.1.3 for Windows (SAS Institute Inc., Cary, NC, USA) statistical software.

Saliva and biofilm samples were collected from both groups at 7 (T_7d_), 21 (T_21d_), 35 (T_35d_), and 49 (T_49d_) days after orthodontic appliance placement. On the 35^th^ day, 1.23% APF gel was topically applied for 4 min after collection of saliva and biofilm samples. At each collection time, biofilm was removed with a sterile probe in a single and continuous movement around brackets of the maxillary right canine (13), maxillary left lateral incisor (22), mandibular left canine (33) and mandibular right central incisor (41) to verify the effect of RMGIC and the topical fluoride application on SM levels.

Biofilm samples were spread on 15x100 mm sterile test tubes containing 4 to 5 glass beads and 2.0 mL phosphate buffer saline (PBS). Saliva and biofilm samples were vortexed for 2 and 1 min, respectively, for microbial desorption, and submitted to ten-fold serial dilutions (10^-5^). After that, 50 mL of each dilution was plated equidistantly on SB-20M culture medium and incubated under candle jar system at 37°C for 48 to 72 hours. The number of colony forming units (cfu) *per* milliliter of saliva and biofilm was counted, and biotyping of colonies with MS characteristics were performed, as describe before for saliva (T_0_).

### Statistical analysis

The original data measured in cfu were transformed in log_10_ for statistical analysis and are reported as log(cfu)/mL. The SM log(cfu)/mL means in saliva before orthodontic treatment (T_0_) and in saliva and biofilm between the two groups at each collection time (T_7d_, T_21d_, T_35d_, and T_49d_) were compared using the Student’s *t* test for independent samples and the Levene test was used to evaluate the homogeneity of variances. The hypothesis of equality of SM log(cfu)/mL means in saliva before treatment (T_0_) and in saliva and biofilm at the different collection times (T_7d_, T_21d_, T_35d_, and T_49d_) was tested using repeated measures ANOVA and Tukey’s test. Data were analyzed using a GraphPad Prism statistical software (GraphPad Software Inc., San Diego, CA, USA) and a significance level of 5% was set for all analyses.

## Results

Analysis of the SM log(cfu)/mL means in saliva at T_0_ by the Student’s *t* test for pairwise comparisons (p=0.42) and the Levene test for homogeneity (p=0.27) showed that the groups were similar.

**Figure 1:**
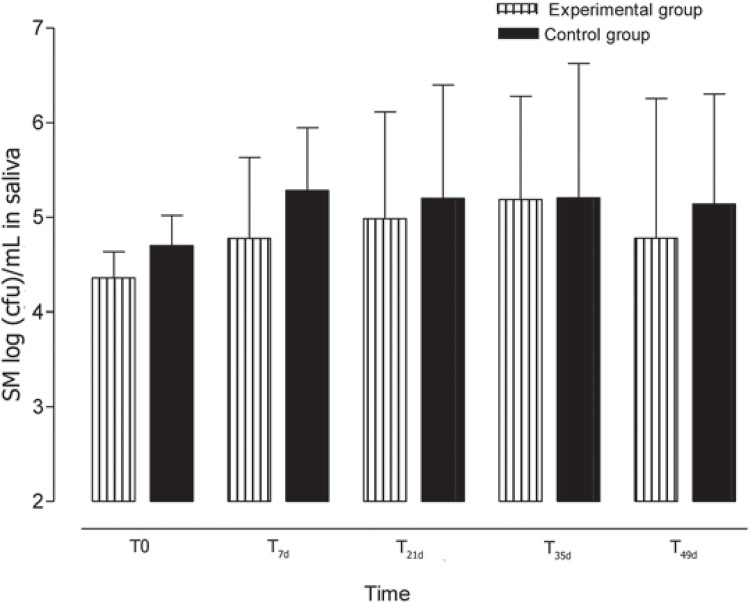
Means and standard deviations [log(cfu)/mL] of *Streptococcus mutans* in saliva for experimental (Fuji ORTHO LC) and control (Transbond XT) groups at different times of collection

Regarding the effect of the tested materials on SM log(cfu)/mL means in saliva, no statistically significant differences were found among the collection times within the Fuji-Ortho (p=0.09) and Transbond XT (p=0.25) groups (Figure 1). Furthermore, no significant differences were found in the SM log(cfu)/mL means in saliva between the Fuji-Ortho and Transbond XT groups at any of the collection times (Table 1). The 1.23% APF gel application at T_35d_ reduced the number of SM log(cfu)/mL in saliva, although not statistically significant.

**Table 1:** Means of *Streptococcus mutans* [log(cfu)/mL] in saliva according to the orthodontic bonding material and sample collection time

	Fuji-Ortho (n=15)	Transbond XT (n=14)	
Collection time	Mean (S.D.)	Mean (S.D.)	p value
T_0_	4.35 (1.03)	4.69 (1.23)	0.42
T_7d_	4.77 (0.85)	5.28 (0.66)	0.08
T_21d_	4.98 (1.13)	5.19 (1.19)	0.62
T_35d_	5.18 (1.09)	5.20 (1.42)	0.97
T_49d_	4.77 (1.47)	4.79 (1.73)	0.97

T_0_ = before bracket bonding. T_7d_, T_21d_, T_35d_, T_49d_ = 7, 21, 35, and 49 days after bracket bonding, respectively. The p values express the results of the Student’s t-test for comparison of the materials at each collection time.

**Figure 2:**
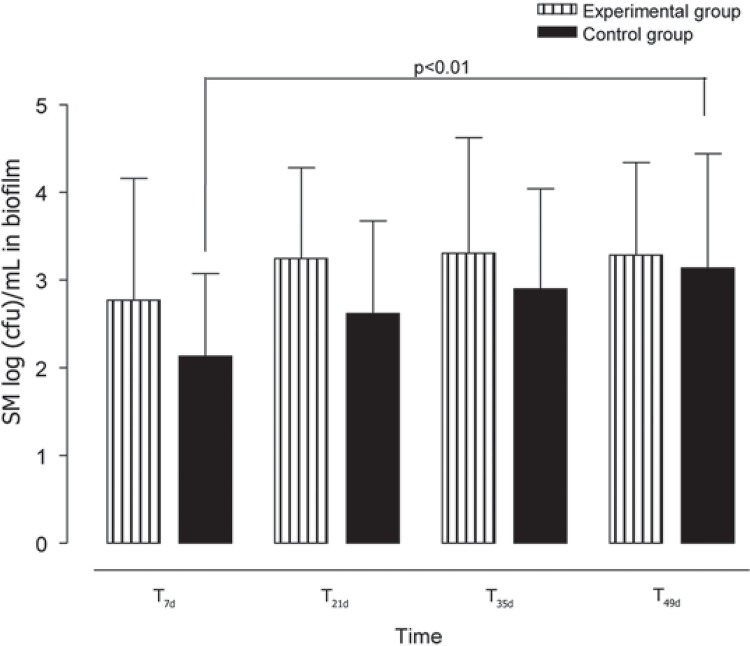
Means and standard deviations [log(cfu)/mL] of *Streptococcus mutans* in biofilm for experimental (Fuji ORTHO LC) and control (Transbond XT) groups at different times of collection

Regarding the effect of the tested materials on SM log(cfu)/mL means in dental biofilm, no statistically significant differences were found among the collection times within the Fuji-Ortho group (p=0.08) although it could be observed a trend of a gradual increase in cfu levels over time (T_7d_ to T_35d_) (Figure 2). In addition, the SM log(cfu)/mL means in dental biofilm numerically remained the same after fluoride application (T_49d_ - Table 2). This gradual increase was also observed in the Transbond XT group, but significant difference was found only between T_7d_ to T_49d_ (p<0.01) (Figure 2). After fluoride application, a numeric increase occurred in SM log(cfu)/mL means in dental biofilm deposits adjacent to the brackets bonded with this material. Comparing the orthodontic materials used, no significant differences were found in the SM log(cfu)/mL means in biofilm between the Fuji-Ortho and Transbond XT groups at any of the collection times (Table 2).

**Table 2:** Means of *Streptococcus mutans* [log(cfu)/mL] in dental biofilm adjacent to the brackets according to the orthodontic bonding material and sample collection time.

	Fuji-Ortho (n=15)	Transbond XT (n=14)	
Collection time	Mean (S.D.)	Mean (S.D.)	p value
T_7d_	2.70 (1.38)	2.13 (0.94)	0.13
T_21d_	3.27 (1.04)	2.61 (1.05)	0.10
T_35d_	3.35 (1.25)	2.75 (1.14)	0.36
T_49d_	3.31 (1.03)	3.13 (1.30)	0.72

T_0_ = before bracket bonding. T_7d_, T_21d_, T_35d_, T_49d_ = 7, 21, 35, and 49 days after bracket bonding, respectively. The p values express the results of the Student’s t-test for comparison of the materials at each collection time.

## Discussion

In this clinical trial, comparisons between two orthodontic bonding materials (RMGIC and composite resin) showed no significant differences in SM cfu counts in saliva and dental biofilm adjacent to the brackets at any collection time. The main difference between the materials was that RMGIC at least allowed a better control of SM cfu counts in dental biofilm hindering the significant increase of these microorganisms along the trial period that was observed in the biofilm adjacent to the composite.

A parallel study design was used to examine the actual effect of fluoride, while most *in vivo* studies on fluoride released by different types of GIC have employed a split-mouth design^11,17,29^. When examining the capacity of fluoride-containing materials to reduce caries onset, it is unlikely that the fluoride released would be confined to only the quadrants in which the test material has been placed and there would inevitably be some crossover effect onto the control side. Although topically applied fluoride has been reported to have mostly a local effect^20^, a slight crossover of fluoride via saliva has also been suggested^10^. This would reduce the difference in effect between the test materials and the power of the experiment to find difference^3,4^. According to Rogers, Chadwick and Treasure^23^(2010), until better understanding how fluoride released on one side of the mouth influences the other side, a parallel study design seems to be the most appropriate.

In this study, no significant differences were found in SM log(cfu)/mL means in the saliva of the patients with brackets bonded with RMGIC and composite resin, and it did not change after topical fluoride application. These results mean that the use of RMGIC to retain orthodontic brackets did not reduce the number of SM cfu counts in saliva. According to Øgaard, et al.^17^ (1997), it could be explained by the fact that the fluoride released by the orthodontic bonding adhesive did not increase the fluoride levels in saliva even when it was associated with a fluoride dentifrice. In the same way, Gorton and Featherstone^9^ (2003) did not find any elevation on salivary fluoride levels, which indicates only a local fluoride release.

Regarding the SM log(cfu)/mL means in dental biofilm deposits adjacent to the brackets bonded with RMGIC, a slight increase was observed from T_7d_ to T_35d_, though without statistical significance. In addition, practically no change occurred in SM log(cfu)/mL means in dental biofilm after topical fluoride application at T_35d_ compared with T_49d_. It can be assumed that RMGIC was not effective in reducing SM counts in dental biofilm adjacent to orthodontic brackets, but no increase in the number of microorganisms was observed either. On the other hand, regarding the SM log(cfu)/mL means in dental biofilm deposits adjacent to the brackets bonded with composite resin, a statistically significant increase was observed from T_7d_ to T_49d_, even after topic fluoride application at T_35d_.

Topical fluoride application was not able to reduce the number of SM along the time of orthodontic treatment, regardless of the material used to retain the brackets, although RMGIC allowed a better control in SM counts than the composite. According to Ahn, et al.^1^ (2011), fluoride-releasing materials can act as a reservoir for topical fluoridation and RMGIC releases the highest amount of fluoride ions when recharged. For this reason, a significant decrease in the number of microorganisms in the biofilm around the brackets was expected for the group that used RMGIC in our study.

The results of this study differ from those of previous investigations. Hallgren, Oliveby and Twetman^12^ (1993) observed significantly higher SM cfu counts in dental biofilm adjacent to brackets bonded with composite (Concise) compared with GIC (Aqua-Cem) 1 month after the beginning of orthodontic treatment. Wright, et al.^29^ (1996) found similar results with significantly lower SM counts in dental biofilm adjacent to GIC (Geristore) than to composite resin (Phase II) 1 week after bracket bonding. After 5 weeks, however, this difference was not detected.

The efficacy of GIC in controlling SM counts in dental biofilm is attributed to the antimicrobial activity of fluoride released from these materials^22,28^, although fluoride release and bacterial inhibition are greater in conventional GIC than in RMGIC^7^.

In agreement to this study, Örthendahl, Thilander and Svanberg^18^ (1997) did not find significant differences in SM cfu counts in dental biofilm adjacent to brackets bonded with GIC (Ketac-Cem) and composite resin (Concise). This result can be attributed to the fact that biofilm samples were collected 9.5 months after bracket bonding. This is an important finding because it has been reported that GIC has a strong antibacterial activity only within the first week, diminishing considerably after this time^1,14^. In the same way, Gillgrass, et al.^7^ (1999) observed that GIC antibacterial activity was significantly higher in the first 24 h.

Several fluoridated materials release high levels of fluoride initially, but the releasing rate drops rapidly and might not be sufficient to prevent caries over the whole course of orthodontic treatment^2,6,8,9^. However, Hallgren, Oliveby and Twetman^12^ (1993) observed that after six months from the beginning of orthodontic treatment the fluoride levels were higher in the biofilm adjacent to GIC than to composite resin.

A material’s ability to be recharged with fluoride or other antibacterial components might extend its antibacterial activity for the duration of the orthodontic treatment. The further development of orthodontic cements to exhibit long-lasting antibacterial properties with fluoride release will minimize the risk of enamel demineralization around brackets^1,6,14^. It has been reported that fluoride release from Fuji-Ortho LC alone fell to minimal values, but with the association of an extrinsic fluoride source, the levels fell initially and then followed an upward trend^5,13,23,24^. These results may be confirmed in this study, in which the SM cfu counts did not increase after 1.23% APF topical application in the RMGIC group, as it occurred in the composite group.

## Conclusion

Considering the results obtained in this study, we may conclude that topical application 1.23% APF gel did not alter SM cfu counts in saliva and dental biofilm adjacent to the orthodontic brackets for both groups; the material used for orthodontic bracket bonding (RMGIC or composite resin) did not alter the SM cfu counts in saliva along the trial period, and the SM cfu counts in dental biofilm adjacent to brackets bonded with RMGIC practically did not change, while there was a significant increase in the SM cfu counts adjacent to brackets bonded with composite resin during the trial period.
